# Effect of Sample Elevation in Radio Frequency Plasma Enhanced Chemical Vapor Deposition (RF PECVD) Reactor on Optical Properties and Deposition Rate of Silicon Nitride Thin Films

**DOI:** 10.3390/ma7021249

**Published:** 2014-02-17

**Authors:** Mateusz Œmietana, Robert Mroczyński, Norbert Kwietniewski

**Affiliations:** Institute of Microelectronics and Optoelectronics, Warsaw University of Technology, Koszykowa 75, Warsaw 00-662, Poland; E-Mails: r.mroczynski@elka.pw.edu.pl (R.M.); nkwietni@interia.pl (N.K.)

**Keywords:** silicon nitride (SiN*_x_*), optical properties, RF PECVD, process parameters, plasma activity, Taguchi’s method

## Abstract

In this paper we investigate influence of radio frequency plasma enhanced chemical vapor deposition (RF PECVD) process parameters, which include gas flows, pressure and temperature, as well as a way of sample placement in the reactor, on optical properties and deposition rate of silicon nitride (SiN*_x_*) thin films. The influence of the process parameters has been determined using Taguchi’s orthogonal tables approach. As a result of elevating samples above the electrode, it has been found that deposition rate strongly increases with distance between sample and the stage electrode, and reaches its maximum 7 mm above the electrode. Moreover, the refractive index of the films follows increase of the thickness. The effect can be observed when the thickness of the film is below 80 nm. It has been also found that when the deposition temperature is reduced down to 200 °C, as required for many temperature-sensitive substrate materials, the influence of the substrate material (Si or oxidized Si) can be neglected from the point of view of the properties of the films. We believe that the obtained results may help in designing novel complex in shape devices, where optical properties and thickness of thin plasma-deposited coatings need to be well defined.

## 1. Introduction

Thin films deposited on various materials and shapes are demanded for number of novel applications. In many cases, the properties of the films do not have to be precisely controlled, especially when the films just protect or passivate the substrate. However, there are also many applications where properties of the films must be controlled in nanometric scale, and what is even more challenging, on objects with complex shapes, including MEMS devices [1], sophisticated photonic devices [2] or optical fibers and sensors [3]. Such capability for uniform deposition on various substrate shapes is offered by the self assembling monolayer (SAM) deposition method, which relies on liquid precursors [4]. The other method offering well-controlled in properties in thin films is atomic layer deposition (ALD) which in turn employs gas precursors [5,6]. Unfortunately, both the methods are relatively time-consuming and limited in materials that can be deposited with them.

In our experiment we have investigated the capability of radio-frequency plasma-enhanced chemical vapor deposition (RF PECVD) in parallel electrode configuration for deposition of thin films on substrates elevated in the plasma reactor which can be later considered as a reference for high objects with different shape, and for those samples where deposition of the film on the bottom surface is expected [3]. The RF PECVD method is well established for standard silicon-integrated-circuit technology where typically flat wafers are used as the substrates. There is an undoubted set of advantages of the method, including, e.g., the possibility of low-cost film fabrication and high efficiency of deposition. The materials can also be deposited as graded-refractive-index films or as a stack of nano-films each with different properties [7]. The growth of the film is due to activation of the gas-phase precursors in a glow-discharge (plasma) environment. The chemical reactions activated by the plasma take place over the substrate, as well as at the substrate. There have been already reported results on uniformity of silicon carbide coating deposited with RF PECVD method on complex shapes placed on the cathode [8]. The difference in thickness of the film deposited on various sides of the coated object has been reported to reach 10%, but the experiment was performed in different electrode configuration and the substrates were placed directly on the cathode.

As a deposited material were chosen silicon nitride (SiN*_x_*) thin films. The SiN*_x_* films have already enjoy wide application in both electronic and optical systems [9–24]. These amorphous films exhibit excellent adhesion to Si and SiO_2_, and stand as a good diffusion barrier against water molecules and sodium ions, two major sources of corrosion and instability in microelectronics [13]. Moreover, the material exhibits good chemical stability and inertness, the qualities which are important in the design of reliable biochemical and biomedical devices [14]. The films also show high values of hardness (~19 GPa) and Young’s modulus (~150 GPa), values that are respectively 2–5 and 3 times higher than those of SiO_2_ [15]. The SiN*_x_* films have a high refractive index, which can be adjusted from that of Si_3_N_4_ (*n*_D_ = 2) to that of amorphous silicon (*n*_D_ = 3.5). Moreover, the films show very low optical absorption in visible and the infrared spectral range. The well-controlled thickness and optical properties of the films deposited on flat surface allowed for their application as an optimum, single-layer antireflecting and protective coating for silicon solar cells [11,16], fabrication of various types of optical waveguides and planar optical systems [7,13,19], and design of novel photonic and optoelectronic devices [2,12,20–22]. In our previous works there has been discussed the application of the SiN*_x_* films as overlays for several types of optical fiber sensors where nano-overlay modifies conditions for light propagation in the structures resulting in tuning of their response [3,23,24]. For such an optical sensing application, the determination of the overlay thickness and optical properties of the films on samples held above the electrode is crucial. In order to improve the uniformity of the coating, the samples must be elevated, allowing for plasma activity all over the samples. When the sample is placed directly on the electrode, physical contact between them makes the deposition on the sample’s bottom less effective.

In this work we discuss the effect of sample elevation in plasma reactor on properties of the obtained films. The investigations are essential for, e.g., precise tuning of the functional properties of new generations of optical devices, such as optical sensors, filters and resonators. Uniform deposition all around the samples require their suspension and simultaneously well defined properties of the overlays are expected. We believe that results of this work can be also useful when design of nano-coated devices on thick substrates or multilayer structures are considered.

## 2. Experimental Details

SiN*_x_* layers were fabricated by PECVD method in an Oxford PlasmaLab System 80+ (Oxford Instruments, Abingdon, UK). The PECVD process takes place in parallel plate RF-plasma (13.56 MHz) reactor where distance between the electrodes is 18 mm. In the course of this work SiN*_x_* films were deposited on p-type silicon and thermally oxidized silicon wafers (oxide thickness 460 nm) in order to investigate influence of substrate material on deposition rate, as well as optical properties of the films. To remove organic and metallic residual contaminants silicon wafers were cleaned prior the processing within standard Radio Corporation of America (RCA) cleaning solutions [25]. After the cleaning, substrates were fully rinsed and immersed in high-purity dionized water. Horiba Jobin-Yvon UVSEL spectroscopic ellipsometer (Horiba Scientific, Edison New Jersey, NJ, USA) with the wavelength ranging from 250 to 750 nm was used to determine the thickness (*d*) and optical properties of investigated SiN*_x_* films, *i.e*., their refractive index (*n*) and extinction coefficient (*k*) [26,27]. To fit the measurement data to a physical model, a three-layer model (Si wafer/SiO_2_/SiN*_x_*) was used where a single-layer Tauc-Lorentz dispersion formula [28] of SiN*_x_* film was applied and fitted with mean-square error χ^2^ < 5.

**Figure 1.**
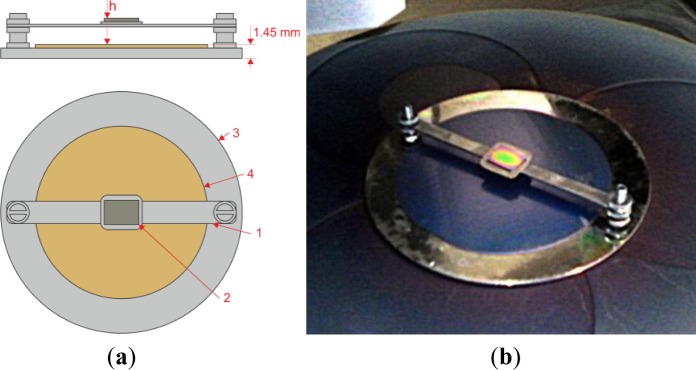
The holder used for elevation of the samples in the reactor, where (**a**) shows its schematic representation with 1: an elevator bar, 2: silicon wafer, 3: stainless steel holder, 4: flattening 3-inch silicon wafer; and (**b**) shows a picture of the holder with a Si sample placed on the elevator bar.

For the purposes of this work a special sample holder was designed which allowed for easy and firm elevation of the wafers in the reactor chamber at precisely adjusted height above the electrode during the deposition process. The sample holder is shown in Figure 1. A 3-inch silicon wafer was placed on the holder substrate for preserving its surface uniformity.

## 3. Results and Discussion

### 3.1. Influence of Deposition Process Parameters

A set of deposition processes was performed first with Si wafers placed directly on the electrode. The aim of this part of the experiment was to find an influence of each input process parameters on *d* and optical properties of the films. In order to minimize the number of the deposition processes, we used Taguchi’s orthogonal arrays approach [29]. Application of the method also allows for simple investigation and analysis of the relations between the input parameters and deposition results. We investigated here the influence of deposition process parameters, *i.e*., the temperature of the electrode (*T*), SiH_4_ and NH_3_ flows (*f*_SiH4_ and *f*_NH3_), and pressure in the deposition chamber (*p*) on *n*, *k* and *d* of the obtained films. The values were determined according to Taguchi’s approach [30]. The 3*^n^* series array applied in this experiment is adopted from [30] where three levels (values) and four factors (parameters) are discussed. Such an approach requires nine experiments to be performed. Responses (results of the experiments) are analyzed by combining the data associated with each level for each factor (column). The difference in the average results for each level is the measure of the effect of that factor. Table 1 shows values of the RF PECVD process parameters applied for each process.

**Figure 2.**
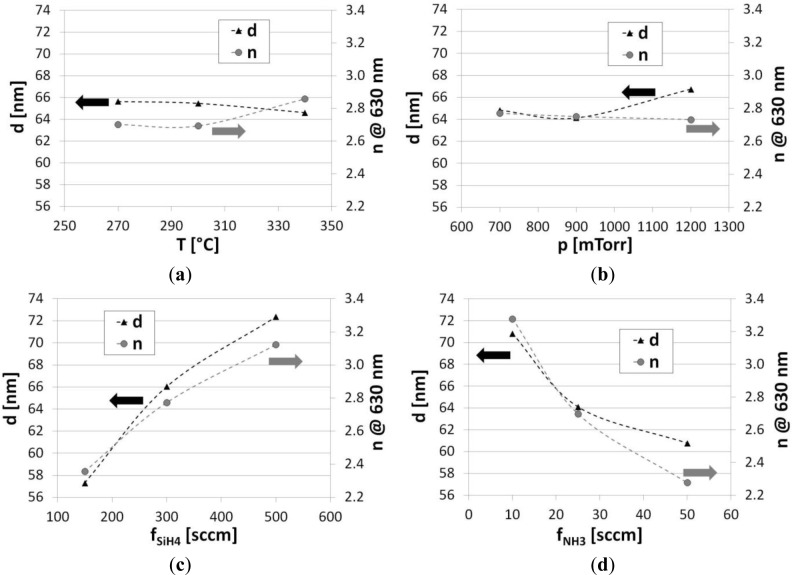
Influence of examined RF PECVD process parameters, *i.e.*, (**a**) temperature; (**b**) pressure; (**c**) SiH_4_ flows; and (**d**) NH_3_ flows, on *d* and *n* (determined at λ = 630 nm), according to the data analysis with Taguchi’s orthogonal tables approach.

After the first set of deposition processes, *d* and optical properties of the deposited SiN*_x_* films have been measured. Spectroscopy ellipsometry measurements have shown that *d* of SiN*_x_* films depending on the process parameters is in the range from 51.1 to 72.5 nm, which corresponds to the deposition rate from 10.2 to 14.5 nm/min, respectively. It has to be emphasized that Figure 2 is prepared according to the analysis of obtained results with Taguchi’s method, and represents only the influence of the deposition parameters on films properties. The analysis has demonstrated that the most significant influence on *d* have *f*_SiH4_ and *f*_NH3_, which cause its increase and decrease, respectively (Figure 2c,d). The impact of other examined process parameters is less significant. The increase of *T* and *p* is followed by slight decrease and increase of *d*, respectively (Figure 2a,b).

**Figure 3.**
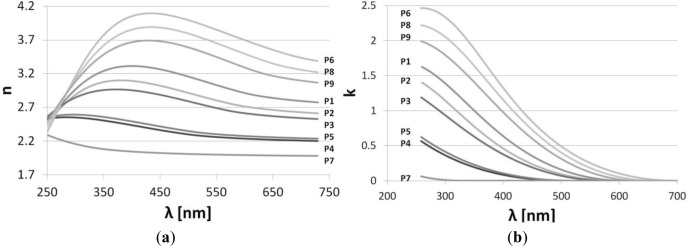
Influence of the deposition process parameters, *i.e*., *T*, *f*_SiH4_ and *f*_NH3_, and *p* determined according to Table 1, on (**a**) *n* and (**b**) *k* of the SiN*_x_* films. The deposition was performed on Si wafers at constant deposition time (5 min) and RF generator power (50 W).

For application of the SiN*_x_* films in optical devices besides *d*, their optical parameters, such as *n* and *k* are of high importance. Therefore, the influence of the process parameters on the optical properties of PECVD SiN*_x_* films has been also examined. Similarly to *d* analysis, in the case of *n*, the most observable influence can be attributed again to reactive gas flows. The relation follows the trends as for *d*. However, the influence of *T* and *p* on *n* is opposite than for *d*, *i.e*., *n* increases with *T* and decreases with *p*. It can be seen in Figure 3 that *k*, which corresponds to optical absorption, follows changes of *n*. For lower *f*_SiH4_ and the higher *f*_NH3_, the value of *n* approaches 2.0 (at λ = 630 nm), which corresponds to *n* of a very good quality LPCVD Si_3_N_4_ layer.

When dispersion characteristics of optical properties are analyzed, high dependence of the optical properties on wavelength can be seen (Figure 3). Interestingly, the analysis of *n* and *k* dispersions has demonstrated that the most uniform value of *n* and the smallest value of *k* in terms of wavelength dependence is characteristic for SiN*_x_* film fabricated in P7 process. This is a very useful and important observation which suggests that the P7 process parameters needs to be chosen for the further investigation of the SiN*_x_* films, especially when they are planned to be used for optical applications.

**Figure 4.**
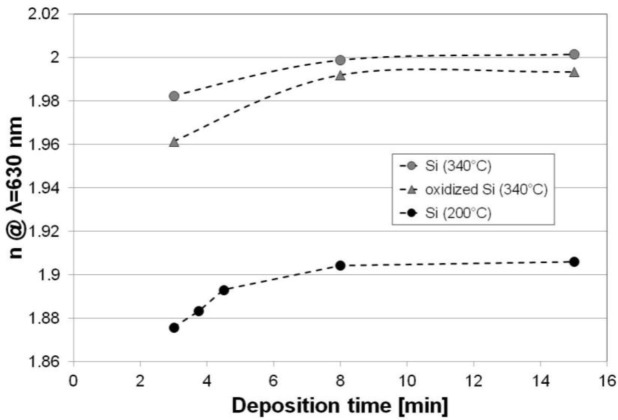
Evolution of the films’ *n* with deposition time for processes performed with *T* of 200 and 340 °C on Si and oxidized Si wafers.

Further investigations have been focused on P7 deposition process parameters set. At first, we varied only *T* and the deposition time, which are the most often key parameters for *T*-sensitive, e.g., polymer containing devices and when *d* of the films matters, respectively. In order to determine the influence of the substrate material, at this stage of the experiment we deposited the films simultaneously on Si and oxidized Si. It has been found that decrease of the *T* from 340 to 200 °C results in significant decrease (by almost 0.1) in *n* of the films (Figure 4). Moreover, films deposited at higher *T* (340 °C) on oxidized Si show slightly lower *n* than those obtained directly on Si wafers. For films deposited at *T* = 200 °C on both Si and oxidized Si wafers, the difference in their *n* is imperceptible (not shown here). Moreover, for the investigated substrates *n* of the films increases with deposition time, especially at initial stage of film growth. For thicker films (*d* > 80 nm), *n* remains independent on deposition time. The phenomenon of *n*(*d*) dependence has been discussed elsewhere [27] for Si-rich SiN*_x_* films and it is likely to be related to densification of the film and releasing of hydrogen with deposition time, as well as stress induced in the film by the substrate at initial stage of the growth. Similar dependence of substrate material on *n* has also been observed for diamond-like carbon deposition and attributed to different thermal and electrical properties of the substrates, corresponding to differences in the density of ions in the plasma above the two surfaces [31]. When the process *T* is lower, the thermal conductivity of the substrate is less important when properties of the film are discussed.

### 3.2. Influence of Sample Placement in the Reactor

**Figure 5.**
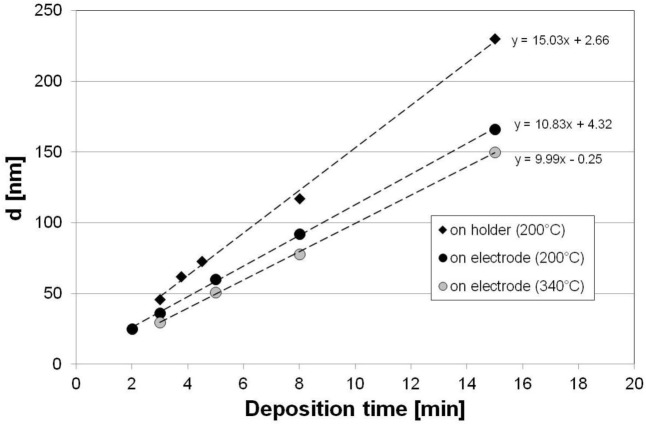
Kinetics of the deposition process *vs. T* and sample placement during the process.

The influence of the placement of the Si wafer in the plasma reactor was investigated next. It can be seen in Figure 5 that *d* increases with the distance between the wafer and the stage electrode. The deposition rate for process taking place on the sample placed on the holder’s surface (1.45 mm above the electrode) is almost 40% higher than the rate when the wafer is placed directly on the electrode. Moreover, the decrease of *T* slightly increases the deposition rate. The *d* measured for films deposited simultaneously on Si and oxidized Si wafers are very similar.

**Figure 6.**
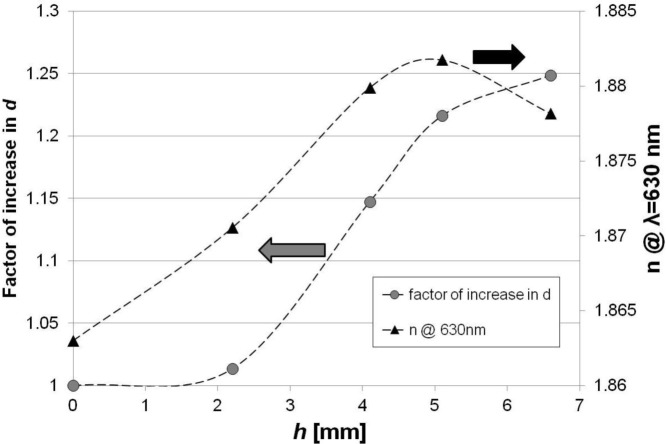
Evolution of *d* and *n* with distance between Si sample and holder. Thickness is referred to the value measured for sample placed directly on the holder.

Since the distance between the electrode and the sample surface is very crucial from the point of view of properties of the films, we placed the sample on a holder’s bar allowing for changing distance (*h*) between the Si wafer and the holder’s surface. The obtained results of *d* and *n* where *d* is referred to the thickness obtained for sample on the holder, is shown in Figure 6. Both *d* and *n* of the films increase with *h*. The dependence is nonlinear, and for the 15 min-long process the increase reaches 17 nm/mm and 3.4 × 10^−3^ RIU/mm in range between 2.2 to 5.1 mm above the holder’s surface for *d* and *n*, respectively. When *h* > 5.1 mm, the increase in *d* is reduced and followed by slight decrease in *n*. In the investigated *h* range, the maximum *d* (25% higher than on the surface) is reached when the sample is held 6.8 mm above the electrode. The decrease in *n* observed for sample held at this level can be induced by lower temperature of the substrate when it is away from the electrode. The influence of temperature on *n* and *d* has been discussed above.

**Figure 7.**
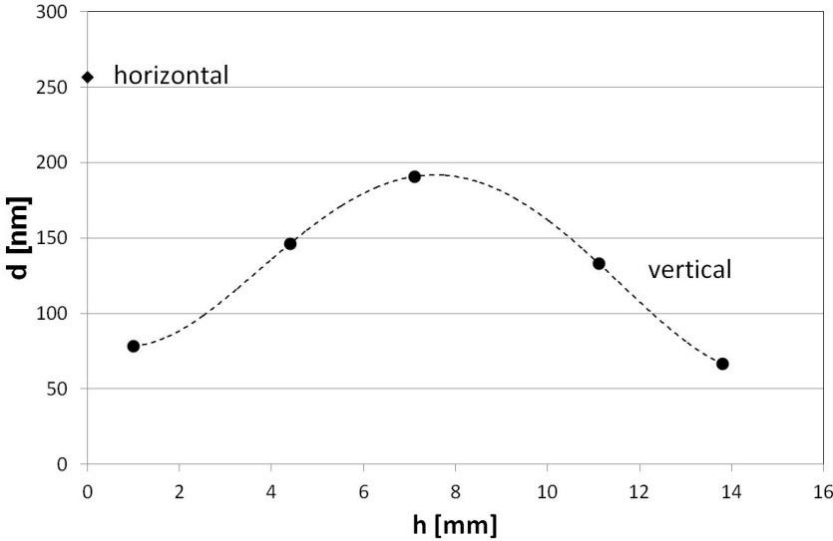
Distribution of *d* on vertically oriented Si wafers. Thickness of the film on wafer horizontally placed on the holder surface is given for comparison.

In order to confirm the effect of increase of the parameters with distance from the electrode, we positioned Si wafer on the holder perpendicularly to the holder’s surface. The process was 15-minutes-long. Thickness of the films on the horizontally (placed on the holder surface) and vertically oriented samples is shown in Figure 7. It can be clearly seen that 7 mm above the surface, the film is deposited as significantly thicker than near to the electrode. The maximum of *d* corresponds well with the results obtained when the elevated sample was investigated. However, in the case of this experiment, the factor of thickness variation referred to as *d* on the sample placed horizontally on the holder reaches 0.75.

We believe that the observed phenomenon is dependent on ion density which seems to be the highest about 7 mm above the electrode. Different volume of the samples placed in the reactor in both the experiments determines *d* and optical properties of the films received each time on the wafers, but the distribution of ion density is similar in both cases. According to [32], structure of both the electric fields and excitation depended on process parameters, but also on topology of the objects in the reactor.

## 4. Conclusions

In the paper we discuss properties of the SiN*_x_* films in dependence on way the sample is placed in the plasma reactor. First of all, using Taguchi’s orthogonal tables approach we have determined the influence of the process parameters on deposition rate and optical properties of the films. Deposition rate increases with SiH_4_ flow and pressure when in turns decreases with NH_3_ flow and temperature. Optical properties of the films, *i.e*., refractive index and extinction coefficient, keep the same trend for gas flows as deposition rate, but the trend is opposite for temperature and pressure. Thanks to the analysis we selected the process parameters resulting in receiving weakly dependent on wavelength refractive index of the films (*n* ≈ 2) and low absorption. Then we noticed that the optical properties depend on thickness of the films, especially when they are thinner than 80 nm. When the temperature is lowered from 340 to 200 °C, as required by some polymer-containing devices, the refractive index decreases, but the influence of the substrate material, *i.e*., Si and oxidized Si, on properties of the films can be neglected.

Secondly, we investigated the influence of the sample placement in the reactor on the properties of the films. We have found that deposition rate increase with the distance from the stage electrode and reaches its maximum about 7 mm above the electrode. The increase in deposition rate is followed by increase of refractive index. The phenomenon must be taken into account when thicker or complex in shape substrates are planned to be coated and where properties of the films in nano-scale are crucial. According to the obtained results, in order to uniformly coat complex in shape surfaces, it is suggested to position the sample surface in the highest plasma activity region, which in our case is located 7 mm above the bottom RF PECVD reactor electrode. Results of this work can be useful when, e.g., MEMS or curved-shape optical structures, including sensors, needs to be uniformly coated with plasma-based method.

## Figures and Tables

**Table 1.** Values of RF PECVD process parameters determined according to Taguchi’s orthogonal tables approach. Process time and power applied to the reactive chamber were constant during all the runs and reached 5 min and 50 W, respectively.

Value/Process	*T* [°C]	*f*_SiH4_ [sccm]	*f*_NH3_ [sccm]	*p* [mTorr]
Value 1	270	150	10	700
Value 2	300	300	25	900
Value 3	340	500	50	1200
P1	270	150	10	700
P2	270	300	25	900
P3	270	500	50	1200
P4	300	150	25	1200
P5	300	300	50	700
P6	300	500	10	900
P7	340	150	50	900
P8	340	300	10	1200
P 9	340	500	25	700
